# Imaging of Metabolic Status in 3D Cultures with an Improved AMPK FRET Biosensor for FLIM

**DOI:** 10.3390/s16081312

**Published:** 2016-08-19

**Authors:** George Chennell, Robin J. W. Willows, Sean C. Warren, David Carling, Paul M. W. French, Chris Dunsby, Alessandro Sardini

**Affiliations:** 1 Cellular Stress Group, MRC Clinical Sciences Centre (CSC), Du Cane Road, London W12 0NN, UK; robin.willows08@imperial.ac.uk (R.J.W.W.); david.carling@imperial.ac.uk (D.C.); 2 Institute of Clinical Sciences (ICS), Department of Medicine, Imperial College London, Du Cane Road, London W12 0NN, UK; a.sardini@imperial.ac.uk; 3 Photonics Group, Department of Physics, Imperial College London, London SW7 2AZ, UK; paul.french@imperial.ac.uk (P.M.W.F.); christopher.dunsby@imperial.ac.uk (C.D.); 4 Centre for Pathology, Department of Medicine, Imperial College London, London W12 0NN, UK; 5 Whole Animal Physiology and Imaging, MRC Clinical Sciences Centre (CSC), Du Cane Road, London W12 0NN, UK; 6 The Kinghorn Cancer Centre, Garvan Institute of Medical Research and St Vincent’s Clinical School, University of New South Wales, Darlinghurst, NSW 2010, Australia; s.warren@garvan.org.au

**Keywords:** FRET, FLIM, AMPK, spheroid, 2-photon, biosensor, TCSPC, 3D culture

## Abstract

We describe an approach to non-invasively map spatiotemporal biochemical and physiological changes in 3D cell culture using Forster Resonance Energy Transfer (FRET) biosensors expressed in tumour spheroids. In particular, we present an improved Adenosine Monophosphate (AMP) Activated Protein Kinase (AMPK) FRET biosensor, mTurquoise2 AMPK Activity Reporter (T2AMPKAR), for fluorescence lifetime imaging (FLIM) readouts that we have evaluated in 2D and 3D cultures. Our results in 2D cell culture indicate that replacing the FRET donor, enhanced Cyan Fluorescent Protein (ECFP), in the original FRET biosensor, AMPK activity reporter (AMPKAR), with mTurquoise2 (mTq2FP), increases the dynamic range of the response to activation of AMPK, as demonstrated using the direct AMPK activator, 991. We demonstrated 3D FLIM of this T2AMPKAR FRET biosensor expressed in tumour spheroids using two-photon excitation.

## 1. Introduction

Genetically-expressed biosensors provide a powerful tool to observe biochemical and physiological changes in live cells and organisms. Such biosensors are particularly useful to monitor metabolism in live cells since biochemical measurements following cell lysis or extraction of mitochondria cannot provide accurate measurements of intracellular parameters or dynamics. Imaging genetically-expressed biosensors can be especially valuable for studying metabolic changes that occur in niche environments, such as the tumour microenvironment, and for rapid changes in response to acute stimulation such as by inhibition of metabolic pathways.

This paper concerns the use of Forster Resonance Energy Transfer (FRET) biosensors expressed in spheroid cultures, which are three-dimensional cell cultures that have been used in recent years to better represent in vivo biology when undertaking in vitro assays since the efficacy of drug candidates in a whole organism can be misrepresented by a screen with conventional 2D (monolayer) cell culture [1,2,3]. Furthermore, 3D cultures based on tumour spheroids may be particularly relevant to the study of anticancer drug responses as these may reproduce key aspects of the tumour microenvironment such as the formation of metabolite and gaseous gradients observed in real tumours [4]. Indeed, cancer drug resistance has been emulated in 3D cultures, highlighting their potential to model disease biology [5]. Heterogeneous responses to drugs have also been observed by changing the cell type in mixed spheroid cultures to reflect different tumour microenvironments and genetically-expressed biosensors have been used to observe distinct responses to the different cells co-cultured in breast cancer cell lines [6].

One drawback of tumour spheroids and other 3D cell cultures is that they are not as straightforward to label or to image as conventional 2D cultures owing to the inherent extension associated with their 3D structure, more complex refractive index profiles, and increased light scattering. It is, therefore, of interest to explore the implementation of assay techniques such as image-based readouts of FRET biosensors in 3D cell cultures with a view to develop more valuable assays, e.g., of drug efficacy. The use of genetically-expressed biosensors, in combination with 3D culture, has been applied to observe redox changes and the onset of apoptosis expected in tumour microenvironments, while exposing the cells to pharmacological agents [6,7,8]. The latter work utilised fluorescence lifetime imaging (FLIM) to read out changes in a FRET biosensor via their impact on the donor fluorescence lifetime. Fluorescence lifetime measurements are inherently quantitative, are independent of fluorophore concentration and are insensitive to attenuation of fluorescence signals by scattering or absorption. For FRET readouts, only measurements of the donor emission are required, making them independent of the spectral variations in attenuation of the sample (or of the instrumentation). Thus, while fluorescence lifetime readouts have many advantages for FRET microscopy generally [9], they are particularly advantageous in complex samples, such as live organisms or 3D cell cultures. In addition, fitting fluorescence decay data to complex decay models has the potential to provide enhanced quantification compared to intensity-based readouts, e.g., providing both FRET efficiency and fraction of FRETing donor. However, the analysis of complex decay profiles requires that the assumptions underlying the chosen decay model are valid for the system being analysed [10], and that experimental factors such as the instrument’s temporal response function are accurately measured and accounted for during analysis. Fit-free approaches, such as the phasor-plot analysis approach [11] avoid the choice of decay model at the initial stage, but still require a model to be chosen when interpreting the phasor plot to obtain quantitative readouts. Optically-sectioned FLIM is most commonly implemented on confocal and two photon excitation (TPE) microscopes using time-correlated single photon counting (TCSPC), which is a photon-efficient method of measuring fluorescence lifetime [12]. Single point scanning confocal and TPE microscopes are limited by photobleaching or phototoxicity within the high peak intensity scanning excitation spot, and this ultimately limits the FLIM image acquisition rate. Multi-spot scanning optically-sectioning FLIM systems can enable faster imaging rates (up to multiple frames per second) by spreading the excitation energy between multiple spots to reduce the peak intensity, and can employ wide-field time-domain [13,14] or frequency-domain detectors [15], or a multi-element time-resolved detector [16]. Marcu et al. [17] provides a relatively recent discussion of the different techniques.

**Figure 1:**
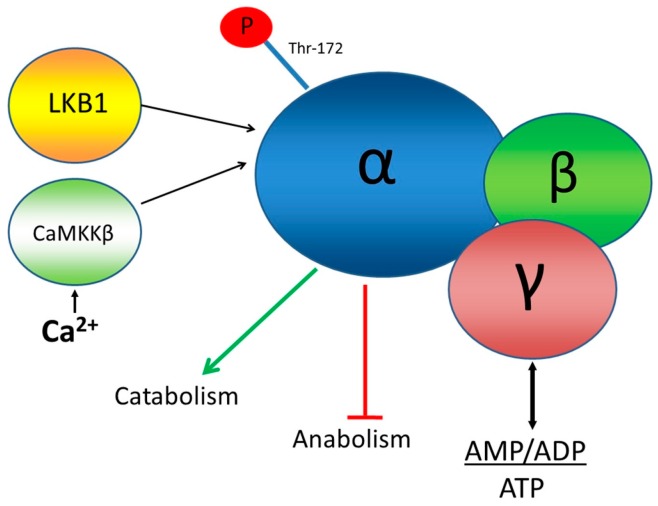
Schematic representation of the structure of AMPK, its regulation and function. Energetic status is sensed by the gamma subunit. The catalytic alpha subunit functions as the effector of this protein complex by phosphorylating target proteins for metabolic regulation.

This paper concerns the application of a novel FRET biosensor for improved Adenosine Monophosphate (AMP) Activated Protein Kinase (AMPK) that has been optimised for FLIM readouts of FRET and which has been evaluated in 2D and 3D cell culture. AMPK is a hetero-trimeric complex that senses the energetic status of a cell by responding to the ratio of AMP + ADP to ATP all of which bind to two Bateman domains of the γ subunit of the complex, as well as to inputs from at least two protein kinases: LKB1, a de facto tumour suppressor, and calmodulin-dependent protein kinase kinase-β (CaMKKβ), as shown in Figure 1. Activation of AMPK by these kinases and AMP or ADP is measured, biochemically, using antibodies for phosphorylated threonine at position 172 in the AMPK α subunit [18]. One of the downstream targets of AMPK, acetyl-CoA carboxylase (ACC) is often used as a measure of AMPK activity. Isoforms of ACC are involved in fatty acid metabolism and are inactivated when they are phosphorylated by AMPK.

AMPK is thought to help control energy expenditure and consumption by inhibiting energy-consuming cellular functions and activate energy-providing functions. A better understanding of the function of AMPK could help elucidate its role in modulating responses to exercise and its activity in diseases, such as diabetes, cancer, and some neurodegenerative diseases, like Alzheimer’s disease.

A FRET biosensor for AMPK, AMPK activity reporter (AMPKAR) has previously been developed by using a substrate recognition site-specific to AMPK, which is phosphorylated at a threonine residue [19]. AMPKAR reports AMPK activity, and not AMPK activation, because it provides a phosphorylatable substrate for AMPK and reflects the balance of the activity of AMPK and phosphatases. The phospho-threonine, when formed is the binding site of the Forkhead Associated 1 (FHA1) domain in the biosensor. When phosphorylated, the AMPKAR conformation changes to produce an increase in the efficiency of FRET from the donor fluorescent protein, enhanced cyan fluorescence protein (ECFP), to the monomeric acceptor fluorescent protein (cpVenus173). The fluorescence emission of ECFP is known to be sensitive to variations in temperature and pH [20], which can alter in different states of metabolism and, so, may produce artefacts, particularly in tumour spheroids where gradients of pH and metabolic activity have previously been reported [4]. The fluorescence decay profile of ECFP is also complex, making FLIM data more challenging to analyse than would be the case with a donor fluorophore presenting a monoexponential decay profile. FRET biosensors have previously been improved by replacing the ECFP donor with mTurquoise2 fluorescent protein (mTq2FP) [21], which is a more photostable cyan fluorescent protein with increased quantum yield and extinction coefficient, a low pKa and a monoexponential decay profile [22]. AMPKAR itself has been modified by replacing the ECFP donor with Cerulean3 fluorescent protein [23], but mTq2FP appears to offer superior performance [22]. This paper presents a novel AMPKAR variant, T2AMPKAR, incorporating mTq2FP as the FRET donor, and its application to FLIM-FRET in combination with two-photon excited fluorescence microscopy in live 3D cultures to visualise the impact of pharmacological interventions.

## 2. Materials and Methods

### 2.1. Imaging Medium

Imaging was undertaken in a medium made of 8.3 g/L Dulbecco’s Minimal Essential Medium (DMEM) powder (Sigma D5030, St. Louis, MO, USA), 10 mM 4-(2-hydroxyethyl)-1-piperazineethanesulfonic acid (HEPES) (Sigma H3375, St. Louis, MO, USA), 1.83 g/L sodium chloride (ICN Biochemicals, Santa Ana, CA, USA), 25 mM glucose (ICN Biochemicals, Santa Ana, CA, USA), 2 mM-glutamine (Gibco 25030-024, Carlsbad, CA, USA), 1 mM sodium pyruvate (Santa-Cruz sc286966, Santa Cruz, CA, USA), 1% *v*/*v* fetal bovine serum (Sigma, St. Louis, MO, USA). The medium is titrated at pH 7.4.

### 2.2. Activator Compounds

AMPK activator 991 [24] is a cyclic benzimidazole derivative. A 100 mM stock solution was prepared in DMSO.

Phenformin hydrochloride (Sigma P7045, St. Louis, MO, USA), a 20 mM stock solution is prepared by dissolving directly into the medium used in imaging experiments.

### 2.3. Cell Culture

HeLa and HEK293T cells were grown at 37 °C in a 5.0% CO_2_ water vapour saturated incubator in DMEM growth medium (Gibco, Carlsbad, CA, USA), supplemented with 10% fetal bovine serum (Sigma, St. Louis, MO, USA).

### 2.4. Imaging Dishes

35 mm diameter glass-bottomed (coverslip of 0.17 mm thickness) Mat-Tek (Ashland, MA, USA) dishes were used for imaging of all samples.

### 2.5. PEI Transfection

Transient transfections were performed by dissolving polyethyleneimide (PEI) (Sigma, St. Louis, MO, USA) with plasmid Deoxyribonucleic Acid (DNA) in a 2.5 µL to 1 µg PEI to DNA ratio in 600 µL of OptiMEM (Gibco, Carlsbad, CA, USA). After 25 min, cells to be transfected were washed twice with PBS and exposed to transfection mix in additional OptiMEM required to cover cells adequately. Transfection mix was removed after 8 h and fresh culture medium was replaced.

### 2.6. Retroviral Transduction and Formation of Clonal Cell Lines

Generation of stable cell clones of the FRET biosensor was achieved by cloning the biosensor gene into pLPC-X retroviral vector by restriction digest with HindIII and EcoR1, followed by ligation and sequencing. A HEK293 cell line with stable expression of the viral Gagpol gene was used as a virus packaging cell, which was transiently transfected with the retroviral biosensor construct and plasmid coding for VSV-g envelope protein. After 24 h, the supernatant of packaging cells was collected, centrifuged to avoid transfer of packaging cells, and placed on target cells with polybrene (Sigma, Dorset, UK). This process was repeated several times over 24 h. Once expression of the biosensor was evident, assessed by observation with an epifluorescence microscope, selection medium containing puromycin (Thermo Fisher Scientific, Boston, MA, USA) was used at a concentration of 2.0 µg/mL. Once selection has occurred after 48 h, 100 cells were plated in a 14 cm Petri dish and allowed to grow for five days. Once colonies were visible, expression of biosensor was assessed with an epifluorescence microscope. Selected colonies were removed using cloning rings and expanded in a six-well plate. Expression of a full length biosensor was assessed by Western blotting and Fluorescence Activated Cell Sorting (FACS) (Figure S4).

### 2.7. Formation of Spheroids

To form spheroid cultures, the Microtissues 12-256 Small Spheroids kit (Microtissues, USA) was utilised to produce 3D Petri dishes. Briefly, agarose was pre-sterilised by heating to 110 °C for 10 min in a dry oven in a suitable vessel. Sterile PBS was then added to constitute a 4% *w*/*v* agarose. When needed, the agarose was melted and 500 µL was dispensed in to the Microtissues mould, cooled and turned out into a well of a six-well plate (Corning). 190 µL of cell suspension was added to a 3D Petri dish and medium was added after 30 min. Spheroids of HEK293T cells typically formed within 24 h and could be used for studies by inversion of the 3D Petri dish and transferred by pipette.

### 2.8. Modification of FRET Biosensor AMPKAR

The pcDNA3.1-AMPKAR plasmid was generously donated by Lewis Cantley (Cornell University, USA). DNA sequences required to generate T2AMPKAR-NES and T2AMPKAR-T391A-NES were designed and ordered from Genscript (Nanjing, China). Once received, plasmids were transformed in XL-10 gold competent cells, amplified and plasmid DNA purified using QIAGEN Plasmid Maxi Kit (Qiagen, Hilden, Germany). Substitution of the AMPKAR FRET donor ECFP with mTq2FP, addition of nuclear export sequence, and introduction of threonine to alanine mutation were achieved by substitution of sequences by restriction enzyme digestions and ligation with appropriate synthesised sequences. DNA sequences were verified by an in-house Sanger sequencing service.

### 2.9. Confocal TCSPC FLIM

Fluorescence lifetime measurements were undertaken using a laser scanning confocal microscope (Leica SP5) using a HCX PL CS APO x63 1.20 NA water immersion objective. Excitation was realised using a mode-locked frequency-doubled Ti:sapphire laser (Mai-Tai, Spectra-Physics), operating at 80 MHz and tuned to 860 nm to obtain an output of 430 nm from a frequency doubler (Inspire Blue—Spectra Physics). FLIM was implemented using a TCSPC module (SPC830, Becker and Hickl, GmbH) for which a trigger signal was taken using a fast photodiode (DET10C, Thor Labs) detecting a portion of the output beam of the Ti:sapphire laser. Fluorescence was detected using hybrid photomultipliers (HPM-100-40, Becker and Hickl, GmbH). A 60 s acquisition time was used for all FLIM images. The instrument response function (IRF) was measured using attenuated excitation light reflected from a coverslip passed directly to detectors without emission filters in place. Analysis was performed using FLIMfit software [25] (Imperial College London, London, UK). Image regions containing cells were first selected using an intensity threshold (integrated photon count >15 per pixel). Cells transfected with donor only were fitted pixel-wise to a monoexponential decay profile, as mTurquoise2 has previously been shown to be well approximated to a monoexponential decay [22]. As expected, we found that the fluorescence decay profiles of the FRET constructs were not well fitted by a monoexponential decay profile and for the experimental acquisition times used, we did not collect sufficient photons for a pixel-wise double exponential fit. Therefore, for the cells expressing FRET constructs, we applied global fitting to a biexponential decay model where the fluorescence lifetime components were determined globally across each image, i.e., with the relative contribution of each decay component being allowed to vary for each pixel. The fluorescence lifetime maps presented report the intensity weighted mean fluorescence lifetime calculated for each pixel from the image-wise global bi-exponential fit.

The global fitting yielded *τ*_1_ = 3.3 ns, *τ*_2_ = 0.7 ns, and *p*_1_/*p*_2_ = 2.3 for the inactive T2AMPKAR-T391A-NES construct and *τ*_1_ = 3.0 ns, *τ*_2_ = 1.2 ns and *p*_1_/*p*_2_ = 1.7 for the active sensor T2AMPKAR in the presence of 50 μM AMPK activator 991, where $p_{i}$ is the pre-exponential factor of the *i*th lifetime component $\tau_{i}$. The differences between donor lifetimes for the two FRET constructs may arise from their different conformations and/or may be a consequence of the FRET measurements averaging over populations of fluorophores that are relatively static during their fluorescence decay, as is the case when using fluorescent proteins [10]. We pragmatically chose to use the mean fluorescence lifetime to provide a read out of the change in activation state of the FRET biosensor.

The contribution of background autofluorescence was measured in untransfected HEK293T cells and included in the fitting model. A 7 × 7 pixel smoothing was applied to the data prior to fitting. The mean and standard deviation of the intensity weighted mean fluorescence lifetime (Equation (1)) was calculated for each image [26]:

(1) $$\langle \tau \rangle =\frac{\sum_{i=1}^{n}p_{i}\tau_{i}^{2}}{\sum_{i=1}^{n}p_{i}\tau_{i}}$$

where 〈τ〉 is the average fluorescence lifetime.

### 2.10. Two Photon Excitation (TPE) TSCPC FLIM

Two photon excitation was implemented with TCSPC FLIM in the same (Leica SP5) laser scanning microscope as above except that the mode-locked Ti:Sapphire laser was tuned to 810 nm and not passed through the frequency doubler. The IRF was measured using gold nanorods (diameter 10 nm, 716820, Sigma Aldrich, St. Louis, MO, USA) dried onto a coverslip [27]. Again, a 60 s acquisition time was used for all FLIM images. Two-photon excitation provides reduced of out-of-plane photobleaching and phototoxicity as well as improved penetration and image contrast when imaging at depth in an optically scattering sample.

### 2.11. Lysis Buffer

Buffer used in Automated Western Blotting and Western Blotting comprised of 50 mM HEPES pH 7.4 (Sigma, St. Louis, MO, USA), 50 mM sodium fluoride (Sigma, St. Louis, MO, USA), 5 mM sodium pyrophosphate (Sigma, St. Louis, MO, USA), 1 mM ethyldiaminetriacetate (EDTA) (Sigma, St. Louis, MO, USA), 10% *v*/*v* glycerol (Sigma, St. Louis, MO, USA), 1% TritonX100 (Sigma, St. Louis, MO, USA) with protease inhibitors benzamidine (Sigma, St. Louis, MO, USA), phenylmethane sulfonyl fluoride (Sigma, St. Louis, MO, USA), and 1 mM dithiothreitol (Sigma, St. Louis, MO, USA).

### 2.12. Automated Western Blotting (WES)

Cells were transferred into treatment solutions 2 h prior to lysis. The lysis procedure consisted of three washes in chilled PBS before final aspiration and addition of lysis buffer. The lysis was performed rapidly to prevent activation of AMPK. Cell lysate was centrifuged at 13,500 rpm at 4 °C for 20 min. The protein concentration of the supernatant was determined by Bradford assay and diluted to 0.6 mg/mL in 10% Wes Sample Buffer (042-195, ProteinSimple, San Jose, CA, USA) and run according to manufactures instructions on a WES capillary electrophoresis western blot machine (Protein Simple). Primary antibodies AMPKβ 1/2 (57C12), rabbit mAb (#4150, Cell Signalling, Beverly, MA, USA), phospho-AMPKα (Thr172) (40H9), rabbit mAb (#2535, Cell Signalling, Beverly, MA, USA), and phospho-acetyl-CoA carboxylase (Ser79) antibody (#3661, Cell Signalling, Beverly, MA, USA) were diluted in WES antibody dilution buffer at 1 in 150, 1 in 150 and 1 in 200, respectively. Peak areas were obtained from the Compass software (Protein Simple V2.5.11) at 30 s exposure. Data from at least two independent samples were used to calculate ratios of peak areas.

### 2.13. Western Blotting

A 15% resolving gel with 5% stacking gel was prepared using Protogel reagents (National Diagnostics, Hessle, Yorkshire, UK) according to manufacturer instructions. Cells on 9 cm dishes at 80% confluency were washed three times with ice cold phosphate buffered saline solution (PBS) and lysed in 600 μL lysis buffer. Lysates were centrifuged at 13,500 rpm for 15 min and the protein concentration of the supernatant was determined by BIORAD assay against BSA standards. Samples were diluted in 5× Laemeli buffer (60 mM Tris-Cl pH 6.8, 2% SDS, 10% glycerol, 5% β-mercaptoethanol, 0.01% bromophenol blue (Sigma, St. Louis, MO, USA)) and heated for 5 min at 95 °C. 12 μg total protein was loaded in each lane of the gel. The gel was run at 175 V at room temperature in SDS PAGE buffer (25 mM Tris, 192 mM glycine, 0.1% *w*/*v* SDS) (National Diagnostics) until the dye front eluted from the gel. The protein was transferred onto methanol-activated PVDF-FL (Immobilon, Millipore, Billerica, MA, USA) membrane at 40 V overnight at 4 °C in transfer buffer (20% methanol, 25 mM Tris, 192 mM glycine). The membrane was blocked in 5% skimmed milk in PBS for 30 min at room temperature. Primary antibodies, Living Colors^®^ mouse anti-GFP a.v. monoclonal antibody (JL-8, Clontech, Mountain View, CA, USA) and Sigma mouse anti-vinculin hVIN-1 (Sigma, St. Louis, MO, USA), were applied overnight at 1:1000 in bovine serum albumin in 50 mM Tris-buffered saline (150 mM NaCl) with 0.05% tween. Secondary antibody IRDye^®^ 680RD goat anti-mouse IgG (LiCOR, Lincoln, NE, USA) was diluted 1:10,000 in 5% skimmed milk in TBST and applied for 30 min at room temperature before imaging on an Odyssey^®^ CLx Imaging System (LI-COR Biosciences).

### 2.14. Flow Cytometry

Cells were resuspended in 2 mM EDTA (Sigma, St. Louis, MO, USA) in PBS and analysed by FACS (Aria Ilu, BD Biosciences, San Jose, CA, USA). For measurement of mTurquoise2 fluorescence, a 405 nm excitation laser was used and emission collected through a 450/50 nm (centre wavelength/bandpass) bandpass filter. For cpVenus173 a 488 nm excitation laser was utilised and emission collected through a 530/30 nm bandpass filter.

## 3. Results and Discussion

The ECFP donor fluorophore of the original FRET biosensor, AMPKAR, has been substituted by mTq2FP in the new biosensor, T2AMPKAR, in order to provide more robust and quantifiable performance for FLIM readouts, in terms of photostability, and reduced sensitivity to environmental factors. It is also important to consider the dynamic range of the biosensor, i.e., by how much the readout signal (donor lifetime) changes upon activation.

In this study we focused our attention to the activity of AMPK in the cytosol. To clarify the ensemble signal from the cells and eliminate the need for more complex segmentation of cellular compartments, we restricted the expression of the T2AMPKAR FRET biosensor to the cytosol by introduction of a C-terminal nuclear export sequence (NES). We employed a leucine-rich nuclear export sequence that was appended to the C-terminus of the acceptor domain of both T2AMPKAR and AMPKAR to simplify analysis.

**Figure 2:**
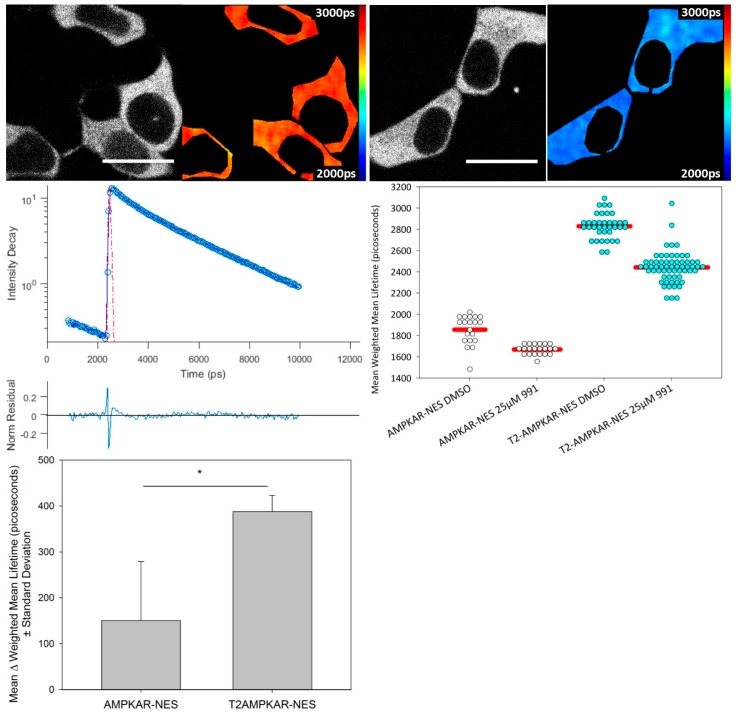
Confocal TCSPC FLIM of AMPKAR-NES and T2AMPKAR-NES. **Top panel**, exemplar intensity images and lifetime maps of T2AMPKAR-NES for both DMSO exposed (**Left**) and 25 µM 991 activated (**Right**) cells are shown; **Middle left panel**, exemplar fluorescence decay profile (blue circles) plotted with double exponential fit to data (blue line), IRF (red dashed line) and residuals (lower); **Middle right panel**, data shown are from three separate experiments. Fluorescence lifetimes for individual cells are shown in dot plot; **Lower left panel**, mean difference in biosensor mean weighted fluorescence lifetime (*n* = 3). Lifetimes are shown in picoseconds (shown in image). Scale bar = 20 µm.

We compared the response of each biosensor using confocal TCSPC FLIM of transiently transfected HEK293T cells using the direct AMPK activator, 991 (Figure 2). The mean donor fluorescence lifetime change was significantly greater for T2AMPKAR-NES (387.92 ± 35.2 ps) compared to AMPKAR-NES (150.7 ± 128.5 ps) (*p* = 0.0184, Student *t*-test, Figure 2 lower left panel). The relative change in donor lifetime for the T2AMPKAR-NES sensor (10%) is larger than that of the original sensor (5%). Note that, for both biosensors, the donor lifetime of the control measurements (for which cells were exposed only to DMSO) is lower than that of the donor alone (3.0 ns ECFP and 3.8 ns for mTq2FP), suggesting that there is FRET under resting conditions.

**Figure 3:**
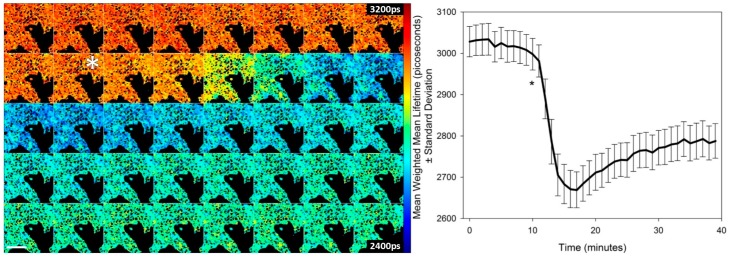
Time course of activation of AMPK by 991. (**Left**) time course montage of confocal TCSPC FLIM maps of T2AMPKAR-NES weighted mean fluorescence lifetimes, an image was acquired each minute; (**Right**) average weighted mean T2-AMPKAR-NES donor fluorescence lifetime in response to addition of 50 µM 991. The asterisk indicates the point of compound addition. Lifetimes are shown in picoseconds (shown in image). Scale bar = 100 µm.

Using a clone of HEK293T cells stably expressing T2AMPKAR-NES we tested the potential of the sensor to report the kinetics of AMPK in response to 991 (Figure 3). HEK293T cells respond rapidly to 991 and, within 15–20 min, a steady state of activation is evident. This steady-state may represent the opposing effects of phosphatases and AMPK on the phosphorylation state of the biosensor.

**Figure 4:**
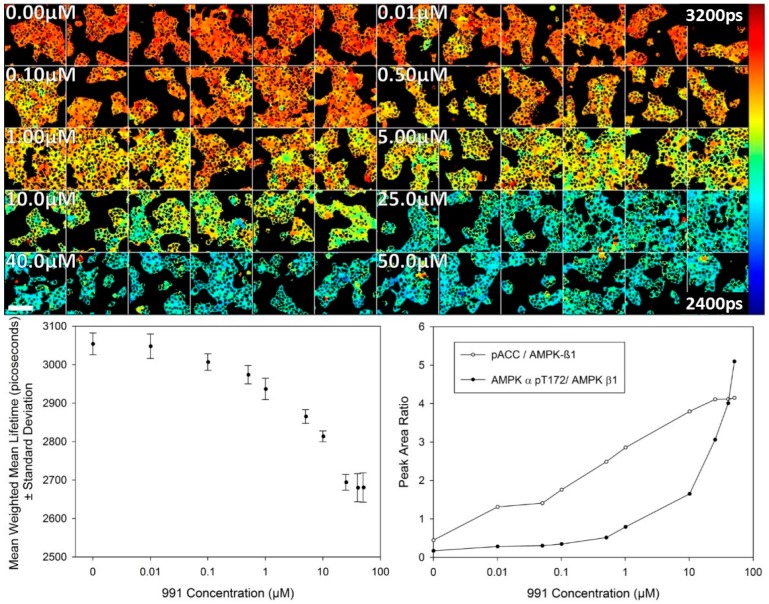
T2AMPKAR-NES dose response to 991. **Upper panel**: montage of confocal TCSPC FLIM maps of the weighted mean lifetime for the dose response; **Lower left panel**: plot of the mean weighted mean lifetime dose response for 991 (*n* = 6); **Lower right panel**: AMPK activity detected by automated Western blotting (WES) of AMPK α phosphothreonine-172, phospho-ACC and AMPK β. Lifetimes are shown in picoseconds (shown in image). Scale bar = 100 µm.

A study of the dose response for activator 991 was then undertaken using FLIM of the T2-AMPKAR-NES biosensor stably expressed in HEK293T cells in 2D cell culture. The variation in donor lifetime as a function of 991 concentration measured 2 h after treatment by confocal TCSPC FLIM is presented in Figure 4. A response to the compound is evident at 100 nM 991 and reaches a maximum response at 25.0 µM as assessed by imaging. For comparison, a dose response curve obtained using a biochemical assay (automated Western blotting) is also presented and shows a similar response (albeit inverted in the vertical direction) due to the fluorescence lifetime decreasing and the peak area ratio increasing with concentration of 991. AMPK-α phosphothreonine 172 is used as a biochemical marker for AMPK activation and is normalised to AMPK β to provide a measure of AMPK activation. Phospho-acetyl CoA carboxylase (pACC) is a marker of AMPK output and is used for comparison here as the biosensor is phosphorylated in a similar fashion as an output of AMPK activity.

In order to verify that the fluorescence lifetime readout of the T2AMPKAR-NES biosensor was not an artefact resulting from changes in the local fluorophore environment, we created a non-phosphorylatable mutant by substituting threonine with alanine at position 391 and then generated a stable cell line of HEK293T expressing this control construct, T2AMPKAR-T391A-NES. The response to 991 of this construct was tested and data are shown in Figure S1. These data indicate that the donor lifetimes of the control construct are not changed by treatment with 991, supporting the conclusion that the lifetime changes observed with T2AMPKAR-NES are due to changes in AMPK activity.

**Figure 5:**
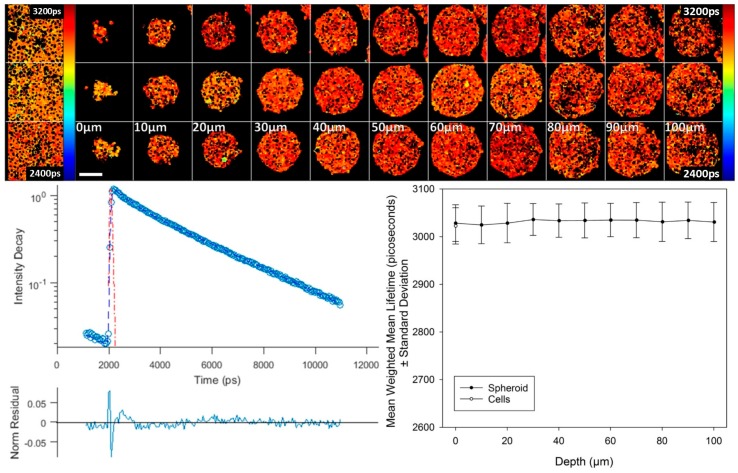
TPE-TCSPC FLIM in spheroids expressing T2AMPKAR-T391A-NES. Top left panel: the weighted mean fluorescence lifetime map for 2D cultures. **Top right panel**: the weighted mean fluorescence lifetime map for three spheroids at different depths (shown in panel); **Left lower panel**: exemplar fluorescence decay profile plotted (blue circles) with double exponential fitting (blue line), IRF (red dashed line), and residuals (lower); **Lower right panel**: plot of the mean weighted mean fluorescence lifetime versus depth. Lifetimes are shown in picoseconds (shown in image). Scale bar = 100 µm.

We also used this control construct to establish that environmental changes in spheroids, e.g., gradients in oxygen, pH or temperature, do not influence biosensor lifetime. Figure 5 shows two photon-excited fluorescence lifetime images throughout 100 µm of depth of three typical spheroids, approximately 250 µm in diameter. These images show a uniform donor lifetime throughout the spheroids. Furthermore, this shows that an equatorial section may be used to faithfully report biosensor fluorescence lifetimes at peripheral and core regions of a spheroid. Similar observations of uniform donor lifetime were found throughout DU145 spheroids expressing mTq2FP alone, as shown in Figure S2. Additionally, Figure 5 shows corresponding fluorescence lifetime images of T2AMPKAR-T391A-NES expressed in 2D cell cultures, which show that the fluorescence lifetime of the donor is similar for the 2D and 3D cell cultures.

**Figure 6:**
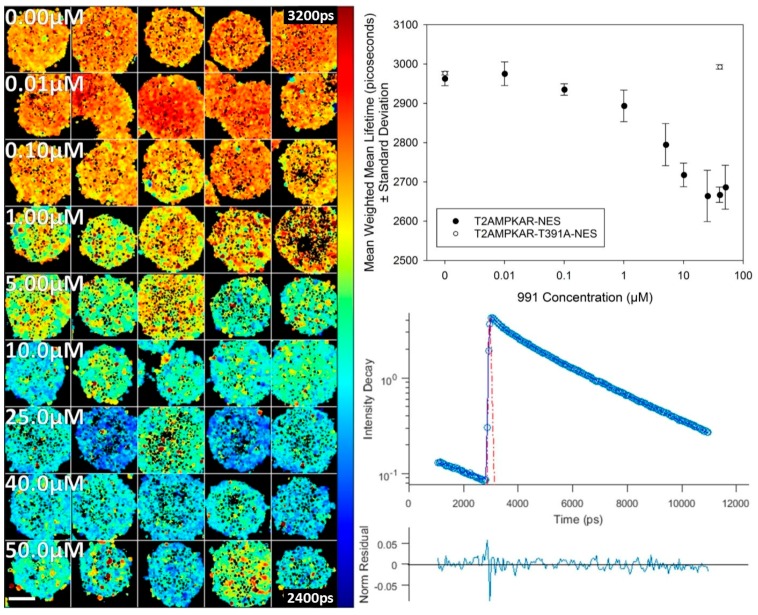
Titration of 991 in spheroids expressing T2AMPKAR-NES using TPE TCSPC FLIM. **Left panel**: FLIM map of weighted mean fluorescence lifetime with increasing concentrations of 991 (shown in panel). **Upper right panel**: plot of the mean weighted mean fluorescence lifetime dose response for 991. Comparison of spheroids expressing T2AMPKAR-T391A-NES is also shown; **Lower right panel**: exemplar fluorescence decay profile (blue circles) plotted with double exponential fitting (blue line), IRF (red dashed line) and residuals (lower). Lifetimes are shown in picoseconds (shown in image). Scale bar = 100 µm.

We then undertook a two photon excited FLIM study of the activation of the T2AMPKAR-NES biosensor in 3D cell culture to compare how AMPK activity changes as a function of concentration of 991. These data, shown in Figure 6, are fluorescence lifetime images through an equatorial plane of the spheroids. They indicate that the dose response in spheroids is similar to that observed in 2D cell culture. For comparison, we also assessed spheroids expressing the control construct which showed no change in donor lifetime upon treatment with 991 (Figure 6 and Figure S3). This experiment shows a uniform activation of AMPK throughout the spheroids and indicates that 991 is permeant to all depths of spheroids.

**Figure 7:**
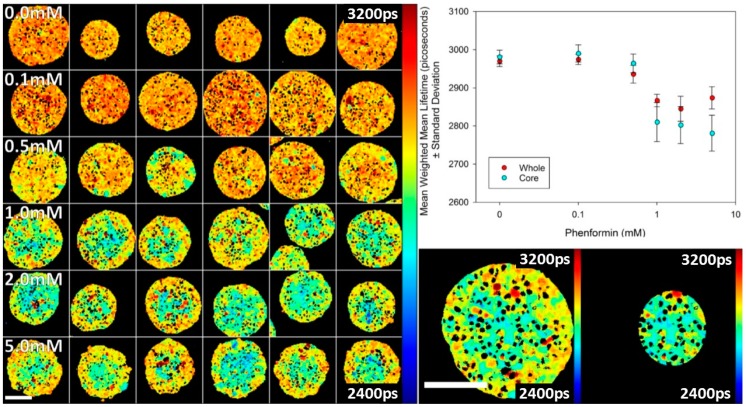
Titration of phenformin in spheroids expressing T2AMPKAR-NES using TPE TCSPC FLIM. **Left Panel**: montage of FLIM maps of the weighted mean fluorescence lifetimes as phenformin concentration is increased (shown in panel). **Upper right panel**: plot of the whole spheroid and core mean weighted mean lifetimes; **Lower right panel**: exemplar images of the whole spheroid and core region segment. Lifetimes are shown in picoseconds (shown in image). Scale bars = 100 µm.

We also studied the effect of an indirect activator of AMPK, phenformin, in spheroids. Phenformin decreases mitochondrial ATP synthesis and, in this way, indirectly activates AMPK [28]. The data shown in Figure 7 indicate that the activation is less uniform than 991. Activation is seen to be greater at the core of the spheroids relative to the periphery. The reason for this behaviour is not yet understood, but may be due to gradients in metabolites, such as glucose, that impact the AMPK activity.

## 4. Conclusions

We have presented T2AMPKAR-NES, a novel variant of the AMPKAR FRET biosensor, which has been specifically modified from the original AMPKAR construct for FLIM. This has been realized by substituting the ECFP donor of the original sensor for mTq2FP. Its advantage has been assessed, in the first instance, in 2D cell cultures where we could detect an increase in sensitivity of the sensor as revealed by an increase of its response to the direct activator of AMPK, 991. Subsequently, we have tested T2AMPKAR-NES in a more optically challenging situation: 3D cell cultures, specifically, spheroids. For this purpose, we have realised HEK293T cell clones expressing T2AMPKAR-NES, as well its non-phosphorylatable mutant, T2AMPKAR-T391A-NES. Spheroids expressing T2AMPKAR-T391A-NES showed a uniform donor lifetime throughout. This allowed us to be confident that our biosensor response was not influenced by spatial variations in the fluorophore environment occurring throughout the spheroids. We proceeded, then, to assess the response to 991 spheroids expressing T2AMPKAR-NES from which a similar dose response to the 2D culture was obtained, suggesting that 991 is able to diffuse so that it is uniformly distributed through the spheroids and uniformly activates the biosensor. Together, our results indicate that our modified biosensor is able to report the activation status of AMPK when read out by FLIM-FRET in combination with two-photon excited fluorescence microscopy in spheroids.

Imaging biosensors in 3D cell cultures enables signalling events to be mapped over space and time, which is important to evaluate the efficacy of therapeutic treatments of diseases in biological tissue—noting that both the tissue and the response to therapy may be heterogeneous. In this paper we have described a FRET probe for reporting the activity of AMPK, a key metabolic sensor, in 3D cultures. Intriguingly, we have reported that the use of an indirect activator of AMPK, phenformin, induced a more prominent activation of AMPK in the core of the spheroids than in the periphery. This may suggest that the spheroids that we have utilized show gradients of metabolites. Although further investigation, outside the scope of this paper, is required to ascertain the nature and function of such gradients, clearly this underscores an important research avenue that the methodology we have described opens.

## Supplementary Materials

The following are available online at http://www.mdpi.com/1424-8220/16/8/1312/s1, Figure S1: Mean fluorescence lifetimes of HEK293T cells expressing T2AMPKAR-T391A-NES exposed to 991; Figure S2: TPE-TCSPC of spheroids of DU145 cells expressing mTurquoise2-NES; Figure S3: Equatorial section FLIM maps of weighted mean lifetime of spheroids expressing T2-AMPKAR-T391A-NES treated with 991 or DMSO; Figure S4: Characterization of HEK293T clones expressing the biosensors.
