# Supplementary material for: Imaging of Metabolic Status in 3D Cultures with an Improved AMPK FRET Biosensor for FLIM
Source: Sensors (Basel). 2016 Aug 19;16(8):1312. doi: 10.3390/s16081312 (PMC5017477; doi:10.3390/s16081312)
Supplement: Supplementary file 1 [file sensors-16-01312-s001.pdf]

Supplementary Materials: Imaging of Metabolic Status in 3D Cultures with an Improved AMPK FRET Biosensor for FLIM

George Chennell, Robin J. W. Willows, Sean C. Warren, David Carling, Paul M. W. French, Chris Dunsby and Alessandro Sardini

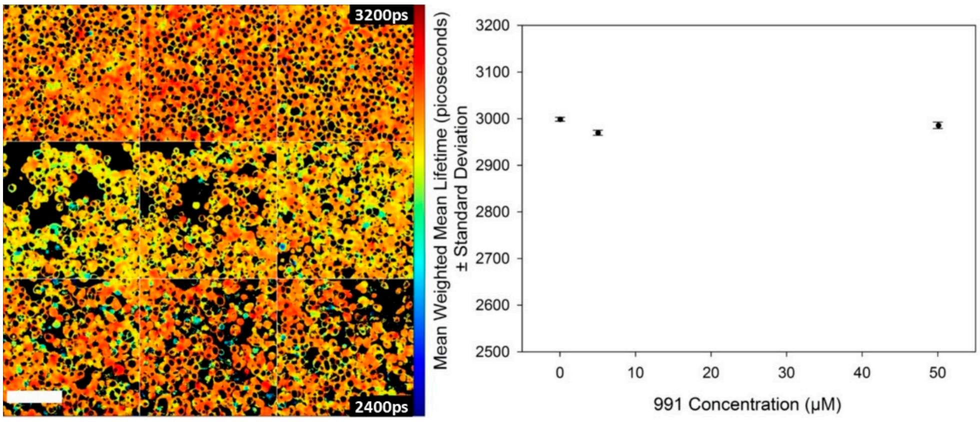

Figure S1. Mean fluorescence lifetimes of HEK293T cells expressing T2AMPKAR-T391A-NES exposed to 991. T2-AMPKAR-NES lifetime does not decrease when 991 concentration is increased indicating that the measured response of T2AMPKAR-NES faithfully reports AMPK activity. Scale bar = 100 μm.

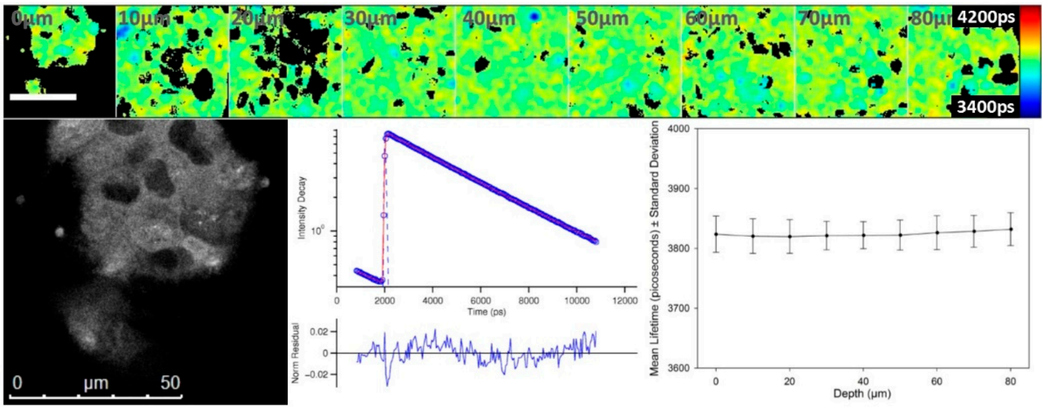

Figure S2. TPE-TCSPC of spheroids of DU145 cells expressing mTurquoise2-NES. Top panel, FLIM map montage of core region of a spheroid at increasing depths (shown in panel). Scale bar = 50 μm. Lower left panel, fluorescence intensity image with scale bar shown. Middle lower panel, exemplar fluorescence decay profile (blue circles) plotted with monoexponential fit (red line), IRF (dashed blue line) and residuals (lower). Lower right panel, plot of mean fluorescence lifetime as a function of depth.

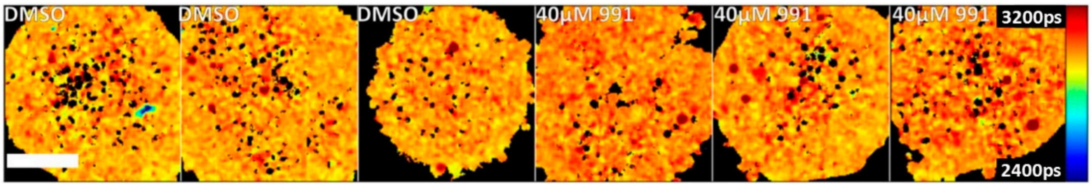

Figure S3. Equatorial section FLIM maps of weighted mean lifetime of spheroids expressing T2-AMPKAR-T391A-NES treated with 991 or DMSO. Scale bar = 100 μm.

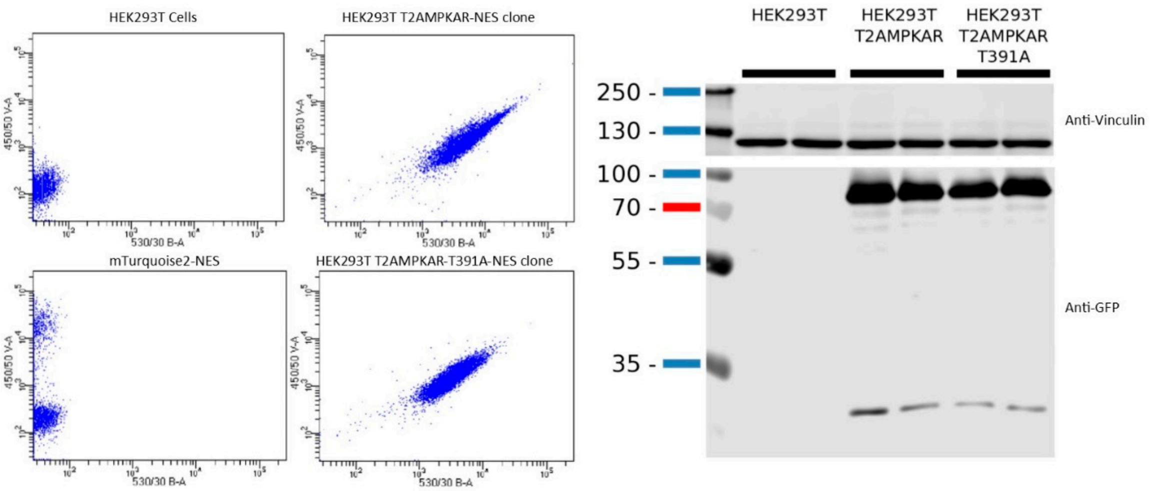

Figure S4. Characterization of HEK293T clones expressing the biosensors. Left panels: FACS detection of fluorescence signal for mTurquoise2 (Y-axis) and cpVenus173 (X-axis) for parental HEK293T cell line, mTurquoise2-NES, T2AMPKAR-NES clone and T2AMPKAR-T391A-NES clone. T2AMPKAR-NES and T2AMPKAR-T391A-NES clones show a single cell population having signals from both fluorophores in a similar ratio. Right panel: detection by Western blotting of expression of the biosensors with an anti-GFP (and its derivatives) antibody. For both T2AMPKAR-NES and T2AMPKAR-T391A-NES clones a predominant band running just above the 70 kDa weight marker is detected (expected molecular weight of the biosensor is 73 kDa). A minor band running below the 35 kDa weight marker is also detected in both clones at similar intensity. Detection of vinculin is used as a loading control.
